# Repetitive transcranial magnetic stimulation for treatment of lactacystin-induced Parkinsonian rat model

**DOI:** 10.18632/oncotarget.17285

**Published:** 2017-04-20

**Authors:** Maowen Ba, Guozhao Ma, Chao Ren, Xuwen Sun, Min Kong

**Affiliations:** ^1^ Department of Neurology, Yantai Yuhuangding Hospital Affiliated to Qingdao Medical University, Yantai 264000, Shandong, PR China; ^2^ Department of Neurology, Shandong Provincial Qianfoshan Hospital, Shandong University, Jinan, 250014, Shandong, PR China; ^3^ Department of Neurology, Yantaishan Hospital, Yantai City, Shandong 264000, PR China

**Keywords:** rTMS, Parkinson's disease, ubiquitin-proteasome system, dopamine, apoptosis

## Abstract

The dysfunction of ubiquitin-proteasome system is an important pathogenesis in the neurodegenerative process of Parkinson's disease. Repetitive transcranial magnetic stimulation (rTMS) is a noninvasive and potential method in treating Parkinson's disease. To investigate whether rTMS has neuroprotective effects in parkinsonian rat model induced by ubiquitin-proteasome system impairment, we gave rTMS daily for 4 weeks to proteasome inhibitor, lactacystin-induced parkinsonian rat model. Rotational behavior test demonstrated that rTMS obviously reduced apomorphine-induced turning number in parkinsonian rats. rTMS could significantly alleviate the loss of tyrosine hydroxylase-positive dopaminergic neurons in lactacystin-lesioned substantia nigra and prevent the loss of striatal dopamine levels. Furthermore, rTMS also reduced the levels of apoptotic protein (cleaved caspase-3) and inflammatory factors (cyclooxygenase-2 and tumor necrosis factor alpha) in lesioned substantia nigra. These results suggest that rTMS can protect nigral dopaminergic neurons against the ubiquitin-proteasome system impairment-induced degeneration by anti-apoptotic and anti-inflammatory molecular mechanism.

## INTRODUCTION

Parkinson's disease (PD) is one common neurodegenerative disease characterized by loss of nigral dopaminergic neurons and the formation of Lewy bodies (LBs) [1, 2]. Substantial evidence has shown that dysfunction of the ubiquitin-proteasome system (UPS) to clear misfolded proteins can play an important role in the pathogenesis of PD [3, 4]. So, new animal models produced by using selective proteasome inhibitors can be valuable for further exploring putative neuroprotective treatment for PD. More importantly, previous research demonstrated that the proteasome inhibitor, lactacystin microinjection into the substantia nigra *pars compacta* (SNc) of rat could cause formation of inclusion bodies resembling LBs and degeneration of dopaminergic neurons [5, 6]. As is known, apoptosis plays an important role in the pathogenesis of PD [7]. Research has also shown an increase in the levels of apoptotic protein caspase-3 within the dopaminergic neurons of lactacystin-induced parkinsonian rat model [8, 9]. Therefore, in this report, we prepared the parkinsonian rat model by injecting one selective proteasome inhibitor, lactacystin into the SNc of rat.

Repetitive transcranial magnetic stimulation (rTMS) is one of the broadly-used, well-tolerated, noninvasive and potential method in the treatment of many neurological diseases, such as PD, focal epilepsy, recovery from stroke, and chronic pain but also psychogenic disorders [10–15]. Research reveals that rTMS has the ability to mediate the neuroplasticity and decrease an imbalance between inhibitory and excitatory inputs from the basal ganglia to premotor and motor zones in PD patients [16–18]. Increased dopamine contents have been observed after rTMS treatment in the striatum of experimental parkinsonian model, and in the anterior cingulate and the orbitofrontal cortices of PD patients [19, 20]. rTMS has also been shown to protect the dopaminergic neurons and improve the rotational behavior in 6-hydroxy-dopamine (6-OHDA)-lesioned parkinsonian rat model [21]. So, It is of great interest to investigate whether rTMS has neuroprotective effects against the proteasome inhibitor-induced dopaminergic neurodegeneration, a model of which may represent progressive condition of PD. Therefore, we designed the following experiment. We explored the efficiency of rTMS treatment, as well as its action mechanisms in treating UPS impairment-induced parkinsonian rat model.

## RESULTS

### Effect of rTMS on apomorphine-induced rotation

**Figure 1:**
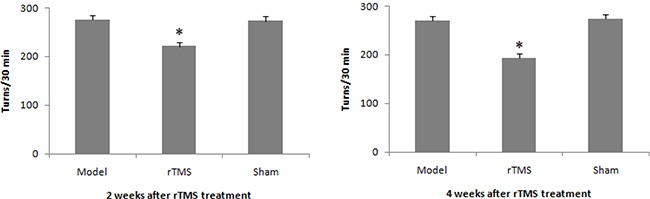
Effects of repetitive transcranial magnetic stimulation (rTMS) treatment on the apomorphine-induced turns in lactacystin-lesioned parkinsonian rats (*n* = 12) Rats were injected subcutaneously with apomorphine (0.5 mg/kg), and the rotation was recorded in 30 min period. *P < 0.05 versus model group and sham stimulation group.

Three weeks after 10 ug lactacystin lesion, the number of turning in model group, rTMS group and sham stimulation group averaged 275.5 ± 5.6, 272.6 ± 8.2, and 269.3 ± 6.5 turns per 30 minutes in the pre-TMS phase, respectively. rTMS treatment attenuated lactacystin-induced rotational behavior after 2 weeks of treatment, and the effects were more obvious after 4 weeks of treatment (Figure 1).

### Effect of rTMS on tyrosine hydroxylase (TH)-positive dopaminergic neurons

**Figure 2:**
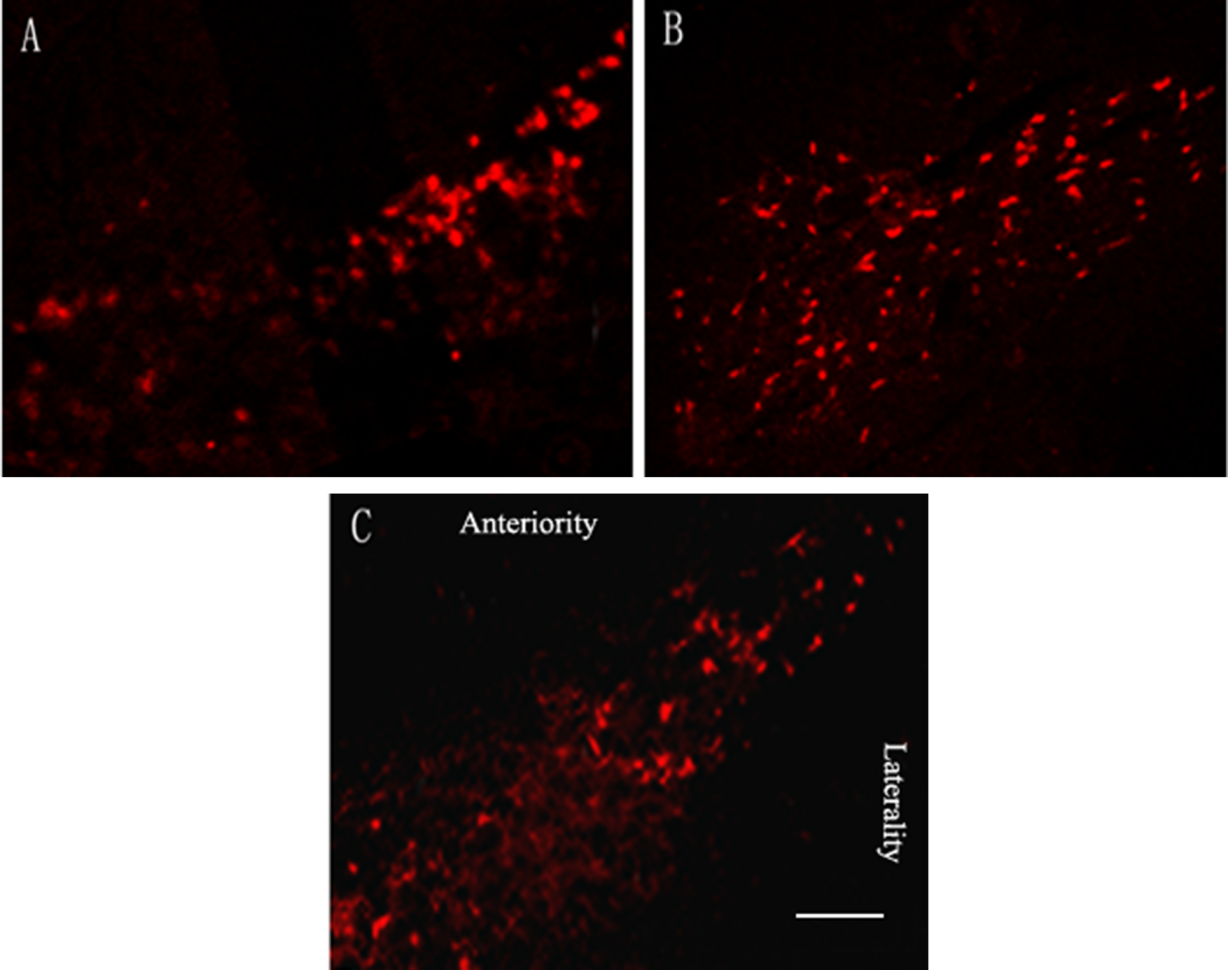
Effects of repetitive transcranial magnetic stimulation (rTMS) treatment on the number of tyrosine hydroxylase (TH)-positive dopaminergic neurons in the substantia nigra of the rats induced by lactacystin (*n* = 6) (**A**) model group; (**B**) rTMS group; (**C**) sham stimulation group. Bar = 0.5 mm.

Microinjection of 10 ug lactacystin into SNc significantly caused TH-positive dopaminergic neuron loss in the ipsilateral substantia nigra. Quantitative analysis showed only 14.8 ± 3.3% nigral TH-positive dopaminergic neurons compared with the control hemisphere. In parkinsonian rats that received rTMS treatment for 4 weeks, there were 37.3 ± 5.6% more TH-positive dopaminergic neurons as compared with sham stimulation group (*P* < 0.05). Sham stimulation has no effect on nigral TH-positive neurons in parkinsonian rats (Figure 2).

### Effect of rTMS on striatal dopamine (DA) levels

**Figure 3:**
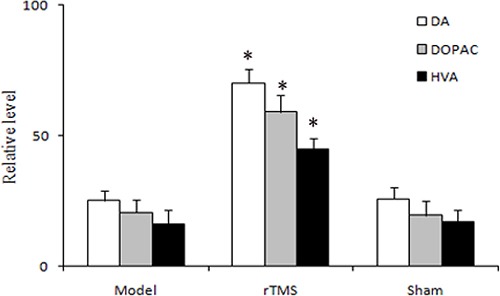
Effects of repetitive transcranial magnetic stimulation (rTMS) treatment on the relative level of dopamine (DA) and its metabolites, 3,4-dihydroxyphenylacetic acid (DOPAC) and homovanilic acid (HVA) in the striatum of the rats induced by lactacystin (*n* = 6) The value of lesioned striatum is expressed as the mean percent of unlesioned striatum. *P < 0.05 versus model group and sham stimulation group.

Biochemical analysis of catecholamine by high-performance liquid chromatography (HPLC) in the striatal tissues was defined as a percentage of the untreated side. The results revealed that lactacystin injection reduced the levels of DA and its metabolites 3,4-dihydroxyphenylacetic acid (DOPAC) and homovanilic acid (HVA) to 25.2 ± 3.7%, 20.7 ± 4.9%, and 16.4 ± 5.1%, respectively. rTMS treatment for four weeks significantly recovered the levels of DA and its metabolites DOPAC and HVA to 70.2 ± 5.3%, 59.2 ± 6.3%, and 45.1 ± 3.9%, respectively (*P* < 0.01, Figure 3). Sham stimulation has no effect on DA and its metabolites DOPAC and HVA in parkinsonian rats.

### Effects of rTMS on the expression of cleaved caspase-3 protein

**Figure 4:**
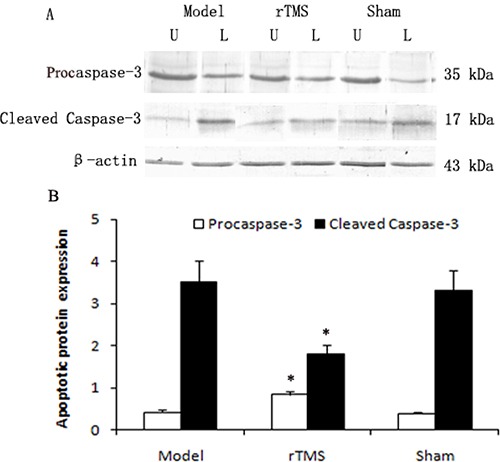
Effects of repetitive transcranial magnetic stimulation (rTMS) treatment on the expression of procaspase-3 and cleaved caspase-3 in the substantia nigra of the rats induced by lactacystin Panel (**A**) The band represents typical immunoblot images detected by antibody against procaspase-3 and cleaved caspase-3 from parkinsonian Model, rTMS and sham stimulation group (*n* = 6). Panel (**B**) Bands corresponding to procaspase-3 and cleaved caspase-3 on immunoblots shown as in Panel A were scanned and their optical density quantified by densitometry and the value of lesioned side expressed as mean ratio of unlesioned side substantia nigra (*n* = 6). U = unlesioned side substantia nigra, L = lesioned side substantia nigra. *P < 0.05 versus model group and sham stimulation group.

Caspase-3 is synthesized as an inactive proenzyme procaspase-3, which is converted into active enzyme, cleaved caspase-3. The level of procaspase-3 and cleaved caspase-3 in substantia nigra was defined as a percentage of the unlesioned side. As demonstrated in Figure 4, the expression of cleaved caspase-3 was upregulated obviously in the parkinsonian model group, and these lactacystin-induced upregulations were clearly reduced in the rTMS treatment group.

### Effects of rTMS on the expression of cyclooxygenase-2 (COX-2) and tumor necrosis factor alpha (TNF-α)

**Figure 5:**
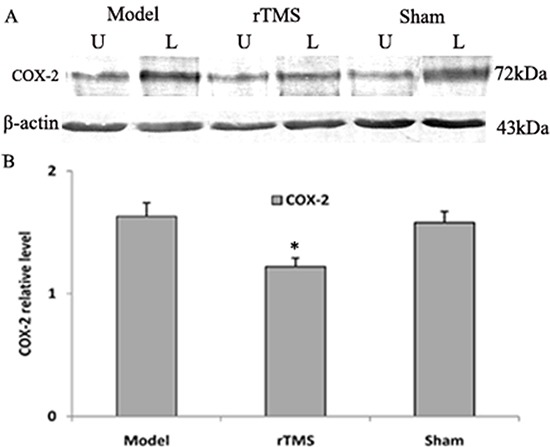
Effects of repetitive transcranial magnetic stimulation (rTMS) treatment on the expression of COX-2 in the substantia nigra of the rats induced by lactacystin Panel (**A**) The band represents typical immunoblot images detected by antibody against COX-2 from parkinsonian Model, rTMS and sham stimulation group (*n* = 6). Panel (**B**) Bands corresponding to COX-2 on immunoblots shown as in Panel A were scanned and their optical density quantified by densitometry and the value of lesioned side expressed as mean ratio of unlesioned side substantia nigra. U = unlesioned side substantia nigra, L = lesioned side substantia nigra. *P < 0.05 versus model group and sham stimulation group.

**Figure 6:**
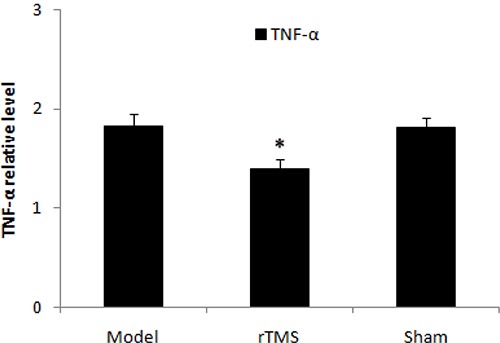
Effects of repetitive transcranial magnetic stimulation (rTMS) treatment on the expression of TNF-α in the substantia nigra of the rats induced by lactacystin The value of lesioned striatum is expressed as the mean ratio of unlesioned striatum (*n* = 6). *P < 0.05 versus model group and sham stimulation group.

The level of COX-2 and TNF-α in substantia nigra was defined as a percentage of the unlesioned side. The expression of COX-2 and TNF-α was upregulated obviously in the parkinsonian model group. These lactacystin-induced upregulations were clearly reduced in the rTMS treatment group (*P* < 0.01, Figure 5 and Figure 6). Sham stimulation has no effect on COX-2 and TNF-α in parkinsonian rats.

## DISCUSSION

PD is characterized with the appearance of motor impairments, which finally respond less to dopaminergic therapy and propose a medical challenge [22, 23]. The non-invasive rTMS has shown promising effects in improving motor disability, and might provide a therapeutic alternative with relatively few side effect [10–13]. In clinical applications, studies performed until now using rTMS displayed heterogeneous results, which can be due to large heterogeneity of cortical targets, stimulation parameters, the variability of patients’ profile, and small sample size, [11, 17, 18, 24]. Future large-sample, well-designed clinical trials are still highly desirable in order to verify the potential therapeutic effect of rTMS. Excitingly, in preclinical study, low-frequency and high-frequency rTMS have been shown neuroprotective effects against in 6-OHDA-lesioned parkinsonian rat model [21, 25]. The possible inadvertent seizures by high-frequency rTMS at high intensities are one of most significant concerns [26]. Low-frequency rTMS did not lead to epilepsy wave on electroencephalograms (EEGs) of rats [27]. The effect of rTMS was also related to stimulation intensity. The present stimulation variables were in accordance with published safety recommendations. The stimulation intensity was set to be above active motor threshold, which could elicit a sustainable effect on cortical excitability without seizures [21]. Therefore, to prevent this side effect, we selected low-frequency (0.5 Hz) rTMS on parkinsonian rats according to previous research [21]. In the period of the experiment, we didn't find any seizure and unpleasantness-like stress behavior in parkinsonian rats. In addition, choosing low-frequency rTMS is also taking into account the need for long-term treatment of PD patients. Then, by use of this well-tolerated rTMS method in the newly lactacystin-induced parkinsonian animal models, we further established the therapeutic effect and action mechanism of rTMS.

In the present study, we found that a 10 ug lactacystin microinjection into SNc lead to significant loss of nigral TH-positive neurons and striatal dopamine content, replicating some of the biochemical and pathological features of PD, which were in agreement with other reports [6]. By use of this model, we further investigated whether low-frequency rTMS treatment could attenuate or reverse the pathological and biochemical changes induced by lactacystin. The low-frequency rTMS treatment started three weeks after the lactacystin lesion and continued for 4 weeks. We found that low-frequency rTMS treatment could provide benefit for behavioral deficit in parkinsonian rats, obviously rescue the loss of nigral dopaminergic neurons and reverse the decrease of striatal dopamine content following lactacystin lesion. These findings suggested that over a relatively short period of time, rTMS treatment can provide adequate therapeutic benefits. The action mechanism of this favourable effects needed further explanation. As is known, previous studies revealed that 20 or 25 Hz high-frequency rTMS could activate the substantia nigra, promote dopamine efflux in terminal zones and thus provide the favourable effects on motor symptoms in parkinsonian rats [28, 29]. Similar to high-frequency rTMS, our study showed that low-frequency rTMS treatment also increased the striatal dopamine concentrations in parkinsonian rats. We believed that low-frequency rTMS prevented TH-positive dopaminergic neurons against being lesioned by lactacystin. Thus, more TH-positive dopaminergic neurons existed and released more dopamine transmitter in the striatum. On the other hand, the present study showed that the increase in striatal dopamine content was more significant than in TH-positive dopaminergic neurons survival after rTMS treatment. Besides the increased numbers of existing TH-positive dopaminergic neurons, the mechanisms of rTMS treatment for PD may involve functional enhancement of residual TH-positive dopaminergic neurons by rTMS, which could release more dopamine transmitter and obviously reduce the number of rotations in parkinsonian rats.

Apoptosis has been proposed to bring about the dopaminergic neuron loss in PD, and caspase-3 may be an ultimate effector. Cleaved caspase-3 is the executor protein of apoptosis, will cut the DNA, and promote cell apoptosis. Inhibition of the caspase family can avoid cell death in a number of models of neurodegenerative cell death *in vivo* and *in vitro*. Previous studies showed that proteasome inhibition can result in apoptosis in TH-positive dopaminergic neurons [7–9]. Caspase-3 was activated in TH-positive dopaminergic neurons after exposure to proteasome inhibitors. As reflected in the present study, proteasomal inhibitor, lactacystin lead to activation of caspase-3, which may contribute to the molecular mechanism of lactacystin-induced apoptosis in nigral TH-positive dopaminergic neurons. The low-frequency rTMS reduced the expression of activated caspase-3 in lactacystin-induced parkinsonian rats, which may be the reason for rescuing nigral degenerative TH-positive dopaminergic neurons.

Neuroinflammation also plays an important role in the pathogenesis of PD [30]. Recent studies have reported that microglial activation and dopaminergic neuron death in substantia nigra were observed in lactacystin-induced parkinsonian model [31, 32]. Lactacystin-induced UPS inhibition can directly trigger neuroinflammation, which leads to neuron death [33]. Previous research has also demonstrated that the expression of various inflammatory molecules increased within the neurons of PD patients [30, 34]. The inhibition of TNF-α and COX-2 has generated neuroprotective effect [35–37]. In the present study, we found that low-frequency rTMS inhibited the expression of TNF-α and COX-2 in lactacystin-induced parkinsonian rat model, and thus prevented dopaminergic neuron apoptosis in parkinsonian rat model.

## MATERIALS AND METHODS

### Animals

Sixty adult Sprague–Dawley rats (female, 200–250 g) were selected for the study. The rats were housed under controlled temperature (21°C) and light conditions (12-h light/dark cycle) with free access to water and standard diet. The protocol relating to animals was approved by the Local Ethics Committee and was carried out in line with the guidelines of the National Institutes of Health for the care and use of laboratory animals (NIH publication No. 80–23) and the Animals Research: Reporting *In vivo* Experiments (ARRIVE) guidelines. The principles of the 3Rs, Replacement, Reduction and Refinement, are incorporated into guidelines and practice of animal experiments in order to safeguard animal welfare.

### Lactacystin-induced parkinsonian rat model preparation

Rats received microinjections of lactacystin. The coordinate of the right SNc was in line with a rat brain atlas (bregma:5.2 mm; lateral:3.2 mm; dura:7.2 mm) [38]. Three weeks after lactacystin microinjections, the rats that displayed apomorphine-induced rotation of over seven turns per minute away from the lesioned side were chosen for the next study [6]. In our preliminary experiment, we tried different dose of lactacystin (2 ug, 10 ug and 15 ug in 2.5 ul physiological saline). The data showed that 10 ug and 15 ug lactacystin had obvious impact on rotational behavior. However, there is no difference of the number of turns between 10 ug lactacystin and 15 ug lactacystin. Thus, a dose (10 ug) of lactacystin was used in the present study. Only saline injection had no impact on rotational behavior of rat.

### rTMS treatment

The thirty-six successfully parkinsonian rats were divided into three groups randomly: model group, sham stimulation group, and rTMS stimulation group (twelve rats per group). In model group, the rats received no stimulation. In rTMS group, the rats received treatment with repetitive magnetic stimulation. rTMS parameters was chosen for the study according to previous research [21]. The rats were placed in the epoxy holder. The magnetic stimulator from BEMS-1 (Shanghai, China) was used. A rodents-coil was placed directly touching the skull of rat that was held gently in a flexible plastic rat restrainer. The parameter of the magnetic stimulations were monophasic pulses, a figure-of-eight coil with the frequency of 0.5 Hz, daily for 4 weeks. Stimulation intensity was adjusted to 250 V/m, which was just above the threshold for evoking motor responses in the hind limb muscles. A stimulation train consisted of 500 pulses. In sham stimulation group, the rats were given sham stimulation, daily for 4 weeks. Sham stimulation was conveyed with the same noise during the rTMS stimulation and separated from the head using a 2 cm plastic spacer cube. This ensured that the rat felt the vibrations produced by the click of the rTMS coil without brain stimulation.

### Behavioral assessment

**Figure 7:**
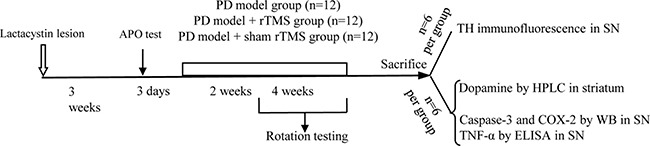
Time-course of the experiments described in the text The effect of repetitive transcranial magnetic stimulation (rTMS) treatment on the lactacystin-induced parkinsonian rat model was studied. APO, apomorphine; TH, tyrosine hydroxylase; COX-2, cyclooxygenase-2; TNF-α, tumor necrosis factor alpha; SN, substantia nigra; HPLC, high-performance liquid chromatography; ELISA, enzyme-linked immunosorbent assay; WB, western blots.

After rTMS treatment, apomorphine-induced rotation (0.5 mg/kg apomorphine, intraperitoneally [i.p.]) was blindly tested in 30 min for each rat to assess the nigrostriatal damage. The rats were placed in a stainless steel bowl. The numbers of rotations in a 30 min test were scored visually for each rat by one blinded rater. The turns of rats were carried out in 2 and 4 weeks after rTMS treatment (Figure 7).

### TH-positive dopaminergic neurons counting by tissue immunofluorescence

Six rats of each group were killed 2 hours after ending the experiments with pentobarbital anesthesia (50 mg/kg body weight, i.p.). Brains were rapidly extracted, then coronal sections through the substantia nigra were cut serially on a freezing microtome at 7 um thickness, collected on gelatin-coated slides. Rat brain sections were preincubated in 10% normal horse serum/0.2% Triton X-100/0.1M phosphate buffer saline (PBS) for 1 h at room temperature. After endogenous peroxidase being quenched with 3% hydrogen peroxide, the sections were incubated with mouse anti-rat tyrosine hydroxylase antibody (1 :1000; Sigma, USA) in PBS containing 1% normal horse serum, washed with PBS, then Rhodamine-binding anti-mouse IgG (1: 400; Vector Laboratories, USA) applied for 1 h, room temperature for 30 min, washed with PBS. Then the results of TH-positive dopaminergic neurons were observed under Axioplan-2 Fluorescence microscopy.

### Dopamine measurements by HPLC

Rats (six rats per group) were sacrificed and striatal dopamine content was detected according to previous report [39]. Results were normalized to wet weight of the sample.

### Caspase-3 and COX-2 protein analysis by western blots

In order to assess the caspase-3 and COX-2 protein expression in the substantia nigra, western blots of the protein extracts (six rats per group) was performed using an primary anti-procaspase-3, anti-cleaved caspase-3 and anti-COX-2 antibody. The method of western blots was performed as follows. 30 mg protein samples taken, 10% sodium dodecyl sulfate polyacrylamide gel (SDS-PAGE) electrophoresis, then transferred to Polyvinylidene-Fluoride (PVDF) membrane, blocked for 1 h with 5% skim milk, and finally closed overnight with primary antibody [(rabbit anti-rat procaspase-3 or anti-cleaved caspase-3 antibody (1:500, Sigma, USA) or anti-COX-2 antibody (1:500, Santa Cruz, CA)(4°C, shaker)], the PVDF were washed 3 times in TBST (TBS with 0.05% v/v Tween-20) at room temperature and then incubated with horseradish peroxidase-conjugated secondary antibody diluted in TBST (1:2,000) for 1 h at room temperature followed by washing, signal detection was performed with an enhanced chemiluminiscence kit, and β-actin as an internal reference, finally analysis of the integral value of the optical density using image analysis software.

### TNF-α analysis by enzyme-linked immunosorbent assay (ELISA)

The supernatants of lysed nigral tissues (six rats per group) were measured for TNF-α concentrations by ELISA protocol in line with the guideline of the manufacturer (Boster, China). Values were transformed to pg/ml according to the standard curve using samples with known TNF-α concentrations. The range of TNF-α values measured by this method was 5–2000 pg/ml.

### Data analysis

Data were presented as mean ± standard error of the mean (SEM). Statistical analysis were processed by analysis of variance (ANOVA) with Dunnett's *t*-test. A *P* value of < 0.05 was regarded statistically significant.

## CONCLUSIONS

In summary, our data demonstrated that in parkinsonian rat model induced by lactacystin, one selective proteasome inhibitor, low-frequency rTMS treatment can be a feasible neuroprotective method to rescue the degenerative dopaminergic neurons. Both anti-apoptotic and anti-inflammatory molecular mechanism are involved in the neuroprotection of low-frequency rTMS.
